# The Serum Metabolome of Moderate and Severe COVID-19 Patients Reflects Possible Liver Alterations Involving Carbon and Nitrogen Metabolism

**DOI:** 10.3390/ijms22179548

**Published:** 2021-09-02

**Authors:** Marianna Caterino, Michele Costanzo, Roberta Fedele, Armando Cevenini, Monica Gelzo, Alessandro Di Minno, Immacolata Andolfo, Mario Capasso, Roberta Russo, Anna Annunziata, Cecilia Calabrese, Giuseppe Fiorentino, Maurizio D’Abbraccio, Chiara Dell’Isola, Francesco Maria Fusco, Roberto Parrella, Gabriella Fabbrocini, Ivan Gentile, Giuseppe Castaldo, Margherita Ruoppolo

**Affiliations:** 1 CEINGE-Biotecnologie Avanzate s.c.ar.l., 80145 Napoli, Italy; marianna.caterino@unina.it (M.C.); michele.costanzo@unina.it (M.C.); fedeler@ceinge.unina.it (R.F.); armando.cevenini@unina.it (A.C.); monica.gelzo@unina.it (M.G.); alessandro.diminno@unina.it (A.D.M.); andolfo@ceinge.unina.it (I.A.); mario.capasso@unina.it (M.C.); roberta.russo@unina.it (R.R.); 2 Dipartimento di Medicina Molecolare e Biotecnologie Mediche, Scuola di Medicina e Chirurgia, Università degli Studi di Napoli “Federico II”, 80131 Napoli, Italy; 3 Dipartimento di Farmacia, Università Degli Studi di Napoli “Federico II”, 80131 Napoli, Italy; 4 Fisiopatologia e Riabilitazione Respiratoria-1 Utsir COVID, Azienda Ospedaliera Specialistica dei Colli-Napoli, 80137 Napoli, Italy; anna.annunziata@gmail.com (A.A.); giuseppefiorentino1@gmail.com (G.F.); 5 Dipartimento di Scienze Mediche Traslazionali, Università degli Studi della Campania “Luigi Vanvitelli”, 81100 Napoli, Italy; ceciliacalabrese123@gmail.com; 6 COVID Unit—Azienda Ospedaliera Specialistica dei Colli—Napoli, Dipartimento di Malattie Infettive ed Urgenze Infettivologiche, 80137 Napoli, Italy; maurizio.dabbraccio@virgilio.it (M.D.); sestadiv@gmail.com (C.D.); francescomaria.fusco@ospedalideicolli.it (F.M.F.); roberto.parrella@ospedalideicolli.it (R.P.); 7 Dipartimento di Medicina Clinica e Chirurgica, Università degli Studi di Napoli “Federico II”, 80131 Napoli, Italy; gabriella.fabbrocini@unina.it (G.F.); ivan.gentile@unina.it (I.G.)

**Keywords:** COVID-19, SARS-CoV-2, coronavirus, metabolomics, mass spectrometry, serum cytokines, lactic acid

## Abstract

COVID-19 is a global threat that has spread since the end of 2019, causing severe clinical sequelae and deaths, in the context of a world pandemic. The infection of the highly pathogenetic and infectious SARS-CoV-2 coronavirus has been proven to exert systemic effects impacting the metabolism. Yet, the metabolic pathways involved in the pathophysiology and progression of COVID-19 are still unclear. Here, we present the results of a mass spectrometry-based targeted metabolomic analysis on a cohort of 52 hospitalized COVID-19 patients, classified according to disease severity as mild, moderate, and severe. Our analysis defines a clear signature of COVID-19 that includes increased serum levels of lactic acid in all the forms of the disease. Pathway analysis revealed dysregulation of energy production and amino acid metabolism. Globally, the variations found in the serum metabolome of COVID-19 patients may reflect a more complex systemic perturbation induced by SARS-CoV-2, possibly affecting carbon and nitrogen liver metabolism.

## 1. Introduction

At the end of 2019, the severe acute respiratory syndrome coronavirus 2 (SARS-CoV-2) has emerged in China as a highly pathogenetic and infectious virus, causing in a short amount of time a worldwide global pandemic [1,2]. The so-called COVID-19 (COronaVIrus Disease 19) is commonly characterized by severe pneumonia and respiratory symptoms, as well as multi-organ failure and death [3,4,5]. Due to the compelling necessity of pharmacological treatments and effective vaccines, drug repurposing represents one of the most tested approaches [6,7,8,9]. The main cause of systemic inflammatory damage is ascribed to the cytokine storm that is depicted by disproportionate release of proinflammatory cytokines leading to lymphocyte exhaustion and a poor outcome [10,11,12,13,14].

Such immune dysregulation is more significant in severe COVID-19 patients [15]. Particularly significant is the relationship between the metabolic regulation and the immune response [16]. The interplay between metabolism and immunity in the host can be explicated by the role of succinic acid as innate immune signal, which can boost the release of IL-1β during inflammation [17]. Furthermore, it was established as a leading function for inflammatory cytokines in host metabolism reprogramming during infections, being able to modulate glucose and lipid metabolism [18].

In the COVID-19 disease, owing to the failure of respiratory functions subsequent to lung damage, the oxygen deprivation involves other tissues and organs such as brain, kidney, and liver. In fact, severe COVID-19 patients may receive mechanical ventilation to supply oxygen deprivation [19]. The modulation of oxygen levels in the organisms is tightly regulated and, in particular, a lactate-induced signaling as response to hypoxia was revealed [20,21,22]. Actually, extremely important is the role of metabolites in oxygen homeostasis, and the association of lactic acid to a waste product of the glycolysis due to the hypoxic environment is common. Despite such considerations, which have represented an axiom for ages, the lactate paradigm has shifted. A lot of evidence suggests that lactate accumulation as consequence of oxygen imbalance is more an exception rather than the rule [20,23]. Nevertheless, it has been shown that lactate modulates the immune response during sepsis [24]. The activated immune cells draw on glycolytic metabolism and the lactate converted during the glycolysis may play an important immunosuppressive role in sepsis [24].

Despite a one-year experience of the pandemic and the advances in understanding the biology of SARS-CoV-2, the mechanisms related to virus infection and disease progression are not exhaustively clear, thus, results are imperative to better investigate the molecular pathways involved in the pathophysiology of COVID-19 [25,26].

In the present study, the serum obtained from a cohort of COVID-19 patients classified according to a different grade of severity as mild, moderate, and severe was used to perform targeted metabolomics analysis in order to detect possible metabolic alterations [27,28,29]. Our analysis defined a clear signature of COVID-19 that includes increased levels of lactic acid in all the forms of the disease. The dysregulation of pathways linked to energy production and amino acid metabolism was disclosed by bioinformatics analysis. Globally, the variations found in the serum metabolome may reflect a more complex systemic perturbation induced by SARS-CoV-2, which might affect carbon and nitrogen metabolism in liver. These lines of evidence speculate on a plausible metabolic reprogramming of the urea cycle and/or Krebs cycle that may control the metabolic responses of the organism to the infection of SARS-CoV-2.

## 2. Results

### 2.1. Serum Metabolomic Alterations in COVID-19 Patients

A targeted metabolomic analysis was performed on serum samples obtained from a cohort of 52 hospitalized COVID-19 patients, classified according to a different grade of severity as mild (*n* = 20), moderate (*n* = 16), and severe (*n* = 16). In addition, 9 control (CTRL) subjects with a negative PCR test for SARS-CoV-2 infection were included in the analysis, with a total of 61 samples analyzed. Globally, 143 metabolites were correctly quantified. A comprehensive list of the measured metabolites, including metabolite names, abbreviations, and group classification, and their raw concentrations in each patient, is shown in Supplementary Table S1.

**Figure 1:**
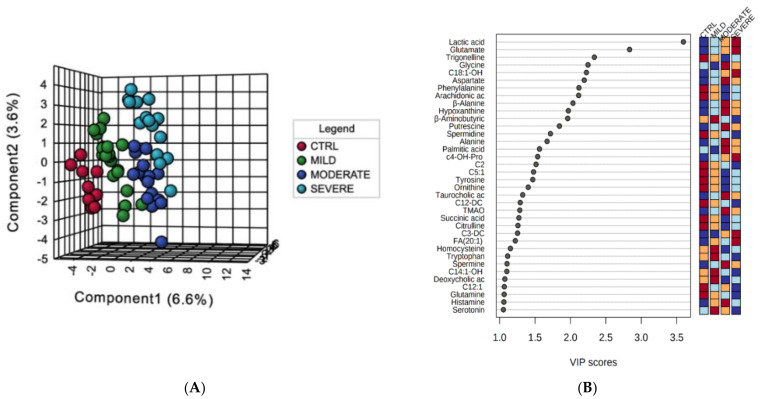

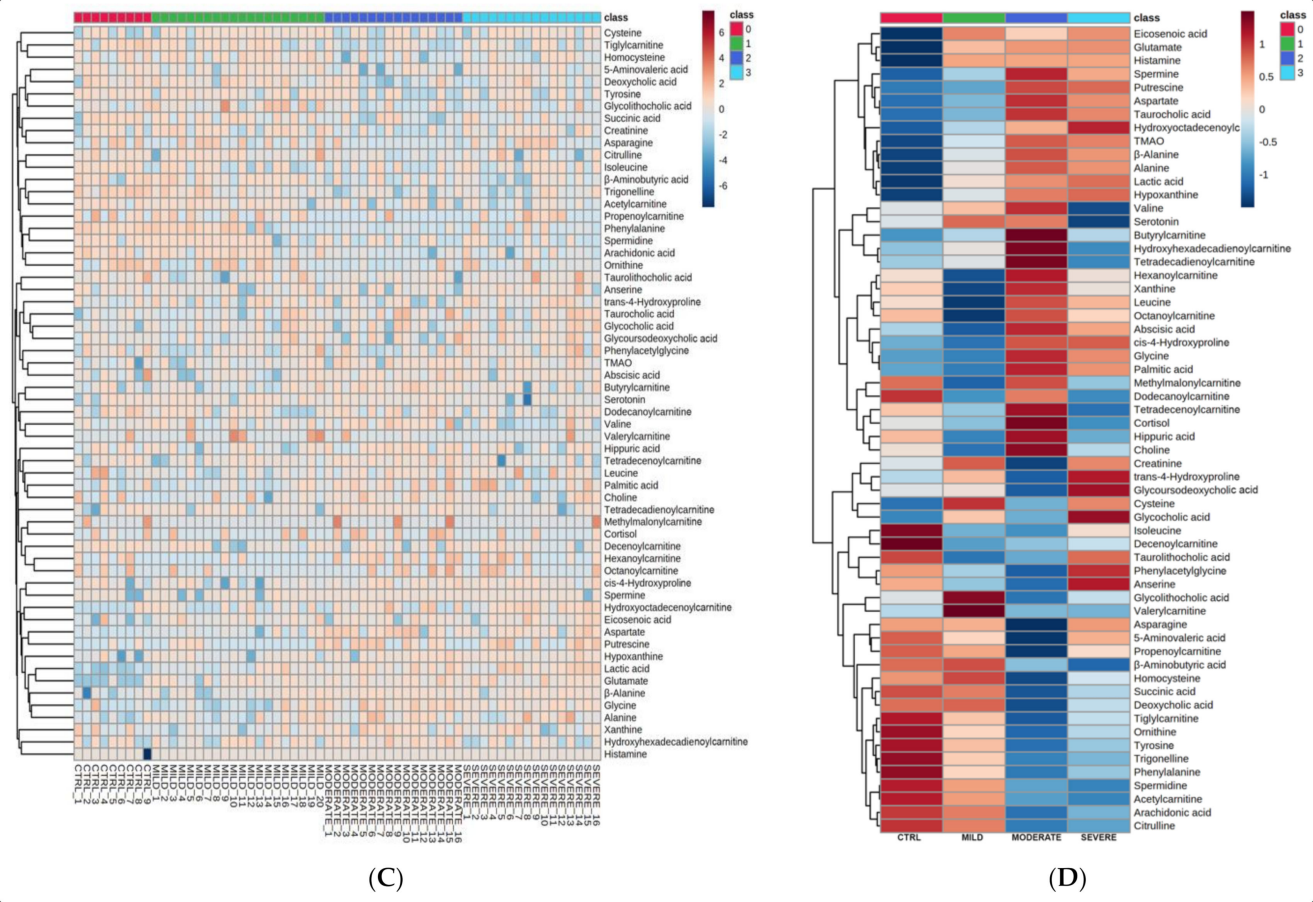
Descriptive changes in the serum metabolome of COVID-19 patients at different degree of severity. (**A**) The supervised partial least squares-discriminant analysis (PLS-DA) plot shows the segregation of the four-condition analyzed metabolomes. (**B**) Discriminant metabolomic features identified according to the Variable Importance in Projection (VIP) score. The 36 most important molecules with values of VIP scores >1.0 are reported. The intensity of the colored boxes denotes the relative metabolite abundance in each group of patients. Heatmaps of the individual (**C**) and average (**D**) serum metabolites concentrations (µM) in each patients group (0 = controls, 1 = mild, 2 = moderate, 3 = severe). The top 60 features ranked by *t*-tests were selected to retain the most contrasting patterns.

To categorize the patients according to the severity of the disease and the metabolic profile, a supervised multivariate analysis was performed. In particular, partial least squares-discriminant analysis (PLS-DA) was carried out to assess the variance between the four analyzed conditions. PLS-DA clustered the metabolome datasets into four well-separated groups, distributing COVID-19 patients according to the disease severity (variance of the principal components (PC): PC1 = 6.6%, PC2 = 3.6%) (Figure 1A). The Variable Importance in Projection (VIP) measure was used to identify the most discriminant metabolites characterizing the four groups, and 36 molecules were highlighted with values of VIP scores >1.0 (Figure 1B). The metabolites with the highest VIP scores were lactic acid and glutamate, both showing an increasing trend in their concentrations from the control and mild groups till the severe condition. Then, the heatmaps of the serum metabolome dataset provided a visualization of the lower and the higher abundant metabolites in each analyzed groups (control, mild, moderate, and severe patients) showing the individual concentrations for each patient and average values (Figure 1C,D).

**Figure 2:**
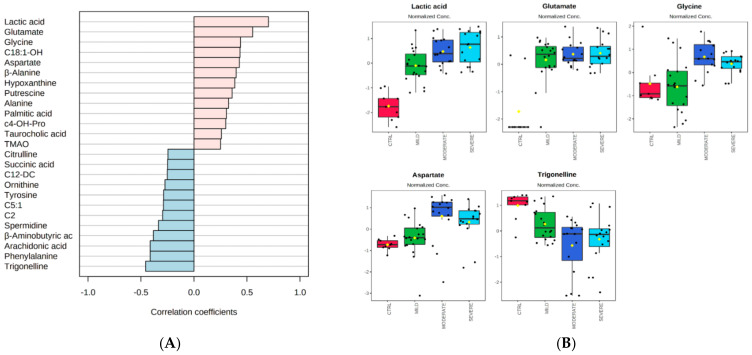
Pattern correlation analysis. (**A**) The analysis was performed correlating the metabolome features with a pattern of concentration increasing toward the degree of COVID-19 severity, in the order from control, to mild, to moderate, to severe patients. The graph reports the significant features detected ordered according to their correlation coefficient. Pink and light blue bars refer to positively and negatively correlated metabolites, respectively. (**B**) The box and whisker plots summarize the normalized values of five selected metabolites, with a correlation coefficient ≳0.5 or ≲−0.5 and lower standard deviations.

Moreover, a correlation analysis of metabolites’ concentrations was performed against a pattern of disease severity, whereas the chosen pattern was used to search for features that increased linearly with the progression of the disease in a time-series data with four severity groups (Figure 2A). According to the VIP analysis, the pattern analysis confirmed a positive correlation (correlation coefficient ≳0.5) of lactic acid and glutamate as the most discriminant metabolites with a crescent pattern from the control/mild to the severe condition, but also of glycine and aspartate; it also revealed a negative correlation (correlation coefficient ≲−0.5) of trigonelline. Metabolites with low correlation values and with high standard deviations were not considered. Graphical plots reporting the normalized distributions of the above-mentioned pattern-correlating metabolites, which all showed in the previous VIP analysis scores >2.0, are displayed in Figure 2B.

**Figure 3:**
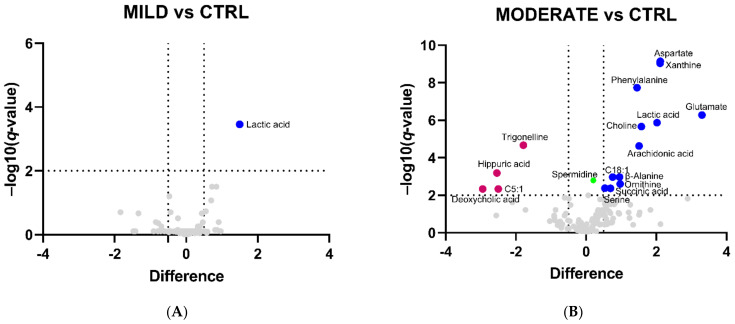

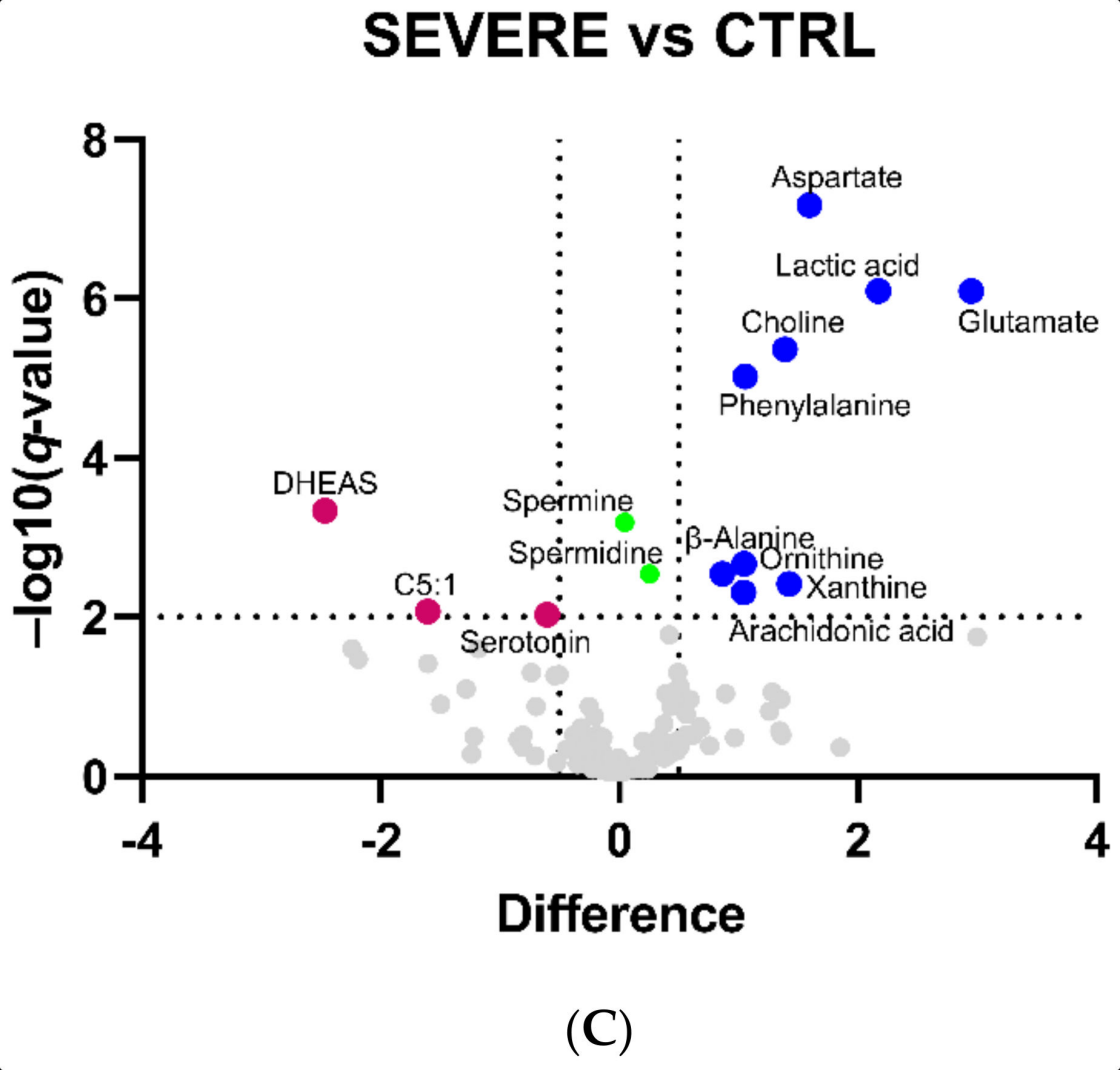
Volcano plot analysis of the differentially abundant serum metabolites in COVID-19 patients. The graphs plot the relative abundance of each metabolite against its statistical significance, respectively reported as difference and −log10 (*q*-value), in (**A**) MILD vs. CTRL, (**B**) MODERATE vs. CTRL, and (**C**) SEVERE vs. CTRL comparisons. Blue and pink dots refer to increased and decreased metabolites, respectively. Green dots refer to statistically significant metabolites with a relative abundance below the difference threshold. Gray dots refer to all the other metabolites identified in the dataset whose relative concentrations are not significantly changed between COVID-19 patients and the CTRL group.

Then, to focus on the independent changes in metabolite levels, univariate statistical analysis was employed to detect more strictly the significant differences in the metabolome profiles, respectively comparing mild, moderate, and severe patients to the control group. Volcano plots for the binary comparisons are reported in Figure 3A–C and a list comprising all the differential metabolites in the three comparisons is shown in Table 1.

The comparison MILD vs. CTRL revealed only lactic acid as a differentially abundant (up-regulated) metabolite, and this difference is maintained in COVID-19 patients also in all the other comparisons, thus suggesting lactic acid dysregulation as the major signature of the serum metabolome of COVID-19 patients. Volcano plot analyses highlighted 16 differential metabolites (4 down and 12 up) in the MODERATE vs. CTRL, and 12 differential metabolites (3 down and 9 up) in the SEVERE vs. CTRL comparison. Spermidine (for both disease groups) and spermine (only for the severe group) were both statistically significant and slightly increased in COVID-19 patients, but those remaining below the considered abundance threshold were not included in Table 1. Revealing a metabolomic signature that brings together moderate and severe COVID-19 patients, we found that the following metabolites commonly increased: lactic acid, glutamate, aspartate (confirming the results obtained from multivariate analysis, Figure 1 and Figure 2), phenylalanine, β-alanine, ornithine, arachidonic acid, choline, and xanthine. On the other hand, C5:1 (tiglylcarnitine) was commonly found decreased. Specific features of the moderate condition are the increase of succinic acid, serine, and C18:1 (octadecenoylcarnitine), and the decrease of trigonelline (confirming multivariate analysis, Figure 1 and Figure 2), hippuric acid, and deoxycholic acid. Specific features of the severe condition are the decrease of serotonin and DHEAS (dehydroepiandrosterone sulfate).

### 2.2. Bioinformatics Enrichment of Dysregulated Pathways

**Figure 4:**
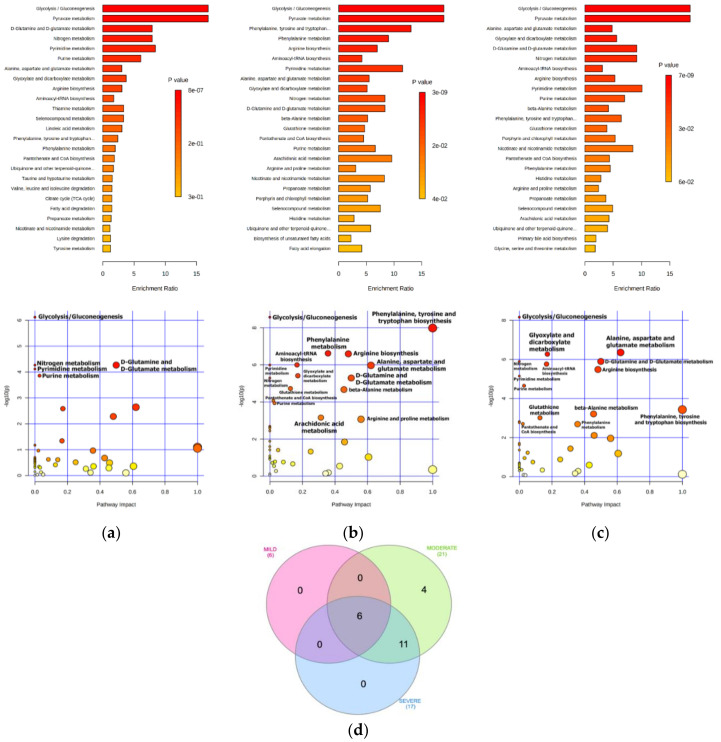
MSEA and pathway analysis in COVID-19 patients. A summary plot for Quantitative Enrichment Analysis (QEA) is reported at the top of the figure showing the top 25 enriched terms for each disease condition: (**a**) mild, (**b**) moderate, and (**c**) severe. At the bottom, the pathway view shows for each condition all the matched pathways according to the *p*-values and the pathway impact values; the size of each pathway is a measure of the number of hits detected within that pathway. For both plots, *p*-values range from yellow (less significant) to red (more significant). (**d**) The Venn diagram shows the relationship between all the pathways identified in mild, moderate, and severe COVID-19 patients highlighting six terms common to all, and 11 terms common to moderate and severe.

To investigate the potential metabolic alterations in COVID-19 patients, combined metabolite set enrichment analysis (MSEA) and pathway analysis was performed in all the three disease comparisons (Figure 4 and Table 2). Reflecting the same level of dysregulation found by volcano plot analysis, bioinformatic analysis revealed more pathways enriched (FDR < 0.01) in the moderate and severe patients rather than mild ones, thus suggesting a lower perturbation of the serum metabolome and related metabolic pathways in patients with fewer symptoms. As is a common signature of the consequence of SARS-CoV-2 infection, the three populations of COVID-19 patients all showed alteration of Glycolysis/Gluconeogenesis (identified also as pyruvate metabolism), d-Glutamine and d-Glutamate metabolism, Nitrogen metabolism, and purine and pyrimidine metabolism. Instead, more metabolic pathways were affected only in the moderate and severe conditions, commonly impacting amino acid metabolism (Phenylalanine, tyrosine and tryptophan biosynthesis, Arginine biosynthesis, Alanine, aspartate and glutamate metabolism, and beta-Alanine metabolism), Glutathione metabolism, Glyoxylate and dicarboxylate metabolism, Nicotinate and nicotinamide metabolism, Pantothenate and CoA biosynthesis, and Aminoacyl-tRNA biosynthesis. The four exclusive pathways enriched in the moderate patients, namely Arachidonic acid metabolism, Arginine and proline metabolism, Propanoate metabolism, Selenocompound metabolism, were also found in the severe condition but were not included because their enrichment was above the FDR threshold.

Above all, the molecular pathways related to the amino acid metabolism, including Phenylalanine, tyrosine and tryptophan biosynthesis, Alanine, aspartate and glutamate metabolism, d-Glutamine and d-Glutamate metabolism, and Arginine and proline metabolism results, were characterized by the most significant pathway impact (Impact score ≥0.5).

### 2.3. Correlation Analyses of Metabolites with Proinflammatory Cytokines in COVID-19 Patients

**Figure 5:**
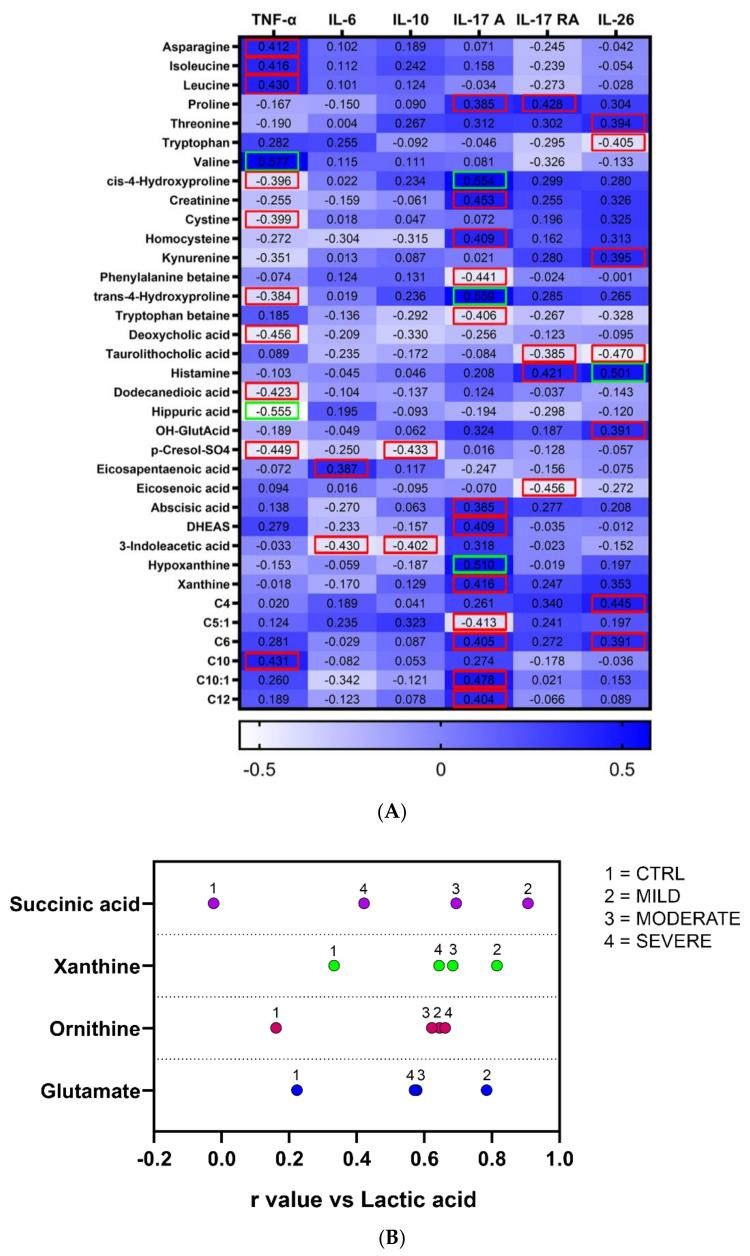
Correlation analysis of the metabolites with inflammatory cytokines and between the metabolites. (**A**) The analysis was performed correlating the concentrations of the metabolites with cytokine parameters in COVID-19 patients. Only metabolites with at least one significant association with cytokines were reported. Dark blue cells denote positive association, while light blue cells denote negative association. Squared numbers refer to significant positive or negative association, with red and green squares indicating *p* < 0.05 and *p* < 0.01, respectively. (**B**) Significant metabolites positively correlating with lactic acid levels in mild, moderate, and severe patients. For each metabolite found statistically significant, a numbered dot corresponding to a given disease group is aligned with the *r* value of correlation with lactic acid on the x-axis.

Correlation analyses were performed to possibly find association between metabolites levels with proinflammatory cytokines, recently described for lung damage and immune response in SARS-CoV-2 infection by leading to local and systemic inflammation [30]. Figure 5A shows the significant correlations obtained by Spearman’s correlation analysis between metabolites and at least one cytokine factor. We found the highest number of associations with the levels of TNF-α, IL-17 A, and IL-26, mostly including amino acids and amino acid-related metabolites. Then, considering lactic acid change as the main signature of the serum metabolome of COVID-19 patients at all the degrees of severity, we found a positive correlation between lactic acid and four molecules, namely succinic acid, xanthine, ornithine, and glutamate, which were found all up-regulated in moderate and severe patients (except for succinic acid that was up-regulated only in the moderate condition). Coherently, these associations were not found significant with the lactic acid levels of the CTRL group (Figure 5B). Furthermore, xanthine was found to positively correlate with IL-17 A.

## 3. Discussion

Our metabolomics study revealed common metabolic signatures affecting all COVID-19 patients and emphasized the major changes to affect the moderate and severe conditions. In particular, our data did not reveal huge alterations in mild patients compared to healthy individuals, suggesting that SARS-CoV-2 infection that induces mild symptoms does not substantially affect the serum metabolome and related metabolic pathways in these patients. Instead, the prevalent differences appear evident in the moderate condition, and are maintained in the severe one. In accordance with the trend of these findings, in a multi-omics study, a high similarity between moderate and severe COVID-19 and a major sharp shift between mild and moderate disease [31] was identified, which would be responsible for the key differences found in the serum metabolome of our patients’ cohort.

Starting from the analysis of the common features, we identified increased levels of lactic acid in all the forms of the pathology, and a crescent quantitative pattern from mild to severe, suggesting this as the major signature of the serum metabolome of COVID-19 patients. Our analysis also revealed the most significantly enriched metabolic pathways in the three populations as Glycolysis/Gluconeogenesis and Pyruvate metabolism, two overlapping terms showing the same enrichment ratio and *p*-value (in each disease subgroup) and both including lactic acid. Even though its quantitative dysregulation did not represent the biggest variation found in comparison with control individuals, this was the only metabolite changing in all the disease forms. Accordingly, lactic acid is one of the factors regularly assured as a serum inflammatory marker, and high levels of lactic acid and lactate dehydrogenase have been identified as strong predictors of COVID-19 disease severity [32,33,34,35].

Lactic acid is a classic marker of mitochondrial metabolic dysfunction together with acylcarnitines [36]. In COVID-19 patients, the mitochondrial energetic mechanisms of ATP production seem to be partially suppressed, suggesting that SARS-CoV-2 infection induces a metabolic shift from aerobic respiration to lactic fermentation (Warburg-like effect) [37]. In addition, increased levels of lactic acid were found in condition of sepsis and circulatory shock. Severe sepsis and such severe inflammatory states are associated with tissue hypoxia, producing high lactate levels that are released from muscular tissues [32,38]. Despite the findings from our analyses, no significant correlation between lactic acid and proinflammatory cytokines levels was identified, but some evidence suggests that there is a pathogenic connection between lactic acid and the immune response, with high lactate levels being strongly associated with a poor outcome and severe adverse effects of COVID-19 [39]. In fact, the novel lactate blockers approach is hypothesized to be potentially beneficial for COVID-19 complications [39,40].

Consistent with our study, also other metabolomics investigations found modification of aspartate, glutamate, alanine, phenylalanine, and arginine amino acid metabolism, revealing increased levels of aspartate, glutamate, phenylalanine, and a decline of serotonin along COVID-19 disease severity, as well as accumulation of succinic acid [18,41,42]. In the mitochondrion, succinic acid can be produced in the metabolic pathway of propionate through the breakdown of branched-chain amino acids, or the β-oxidation of odd-chain fatty acids and cholesterol degradation [43,44]. The increase of such amino acids and succinic acid might be related to a dysregulation of the liver central carbon metabolism in COVID-19 patients and to general metabolic and oxidative stress [41]. For example, the inflammation induced by severe sepsis and the increase of ROS (reactive oxygen species) levels deplete a significant amount of tetrahydrobiopterin, which is the cofactor of the phenylalanine hydroxylase for the conversion of phenylalanine to tyrosine in hepatocytes, limiting phenylalanine metabolization. Thus, serum levels of phenylalanine showed augmentation in such patients as a result of the activation of the immune response, also magnifying inflammation processes [45,46,47]. The activation of the immune response and inflammation is observed also in other diseases or models of metabolic disorders showing impairment of amino acid metabolism, in which cytokines’ release is strictly regulated by the inflammasome [48,49,50]. In addition, being succinic acid, an innate immune signaling molecule during inflammation in macrophages, its increase may enhance the cytokine production during the cytokine storm syndrome that affects COVID-19 patients [18].

**Figure 6:**
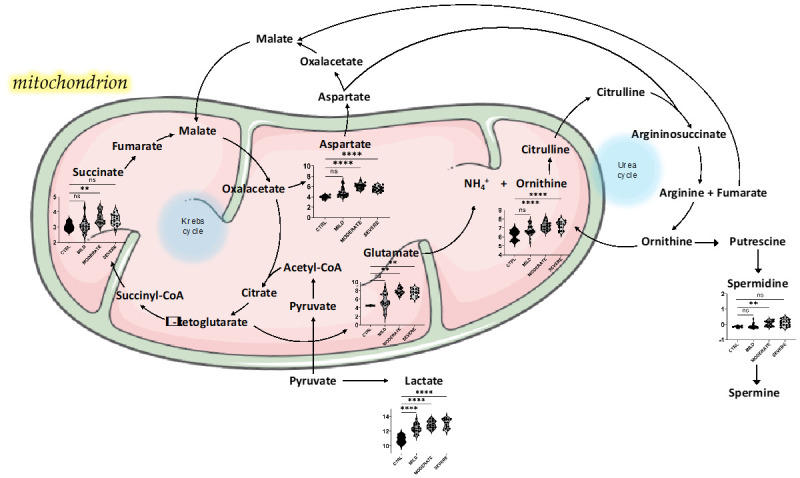
Proposed model for a plausible liver metabolic perturbation upon SARS-CoV-2 infection that involves energy pathways and nitrogen metabolism. The metabolites reported in the figure are interconnected within the mitochondrial Krebs and urea cycles. Metabolite violin plots were reported only for significantly changed metabolites between CTRL and the diverse forms of pathology as resulted from volcano plot analysis. The violin plots show log2-transformed and normalized concentration values analyzed by one-way ANOVA with Dunnett’s multiple comparison test correction. Significant * are all below 95% confidence (** *p* < 0.01, **** *p* < 0.001). NS = not significant. This figure was drawn adapting the vector image form the Servier Medical Art bank (http://smart.servier.com/; last accessed 24 June 2021).

We found that the increase of serum lactic acid strongly correlates in a positive trend with the significant increase of succinic acid, but also xanthine, ornithine, and glutamate in all mild, moderate, and severe COVID-19 patients. This suggests a strong connection between the hypoxemia state and the subsequent oxidative stress and the dyslipidemia of COVID-19 patients, which may have a paramount effect on the mitochondrial energy metabolism and detoxification processes in the liver [30,41]. Our metabolomics data sharply suggest impairment of the metabolic hub that connects the urea cycle and the Krebs (Tricarboxylic acids, TCA) cycle (Figure 6).

Despite many reports highlighting liver injury as associated with coronaviruses infection, possibly resulting from a direct insult or as part of the systemic inflammatory reaction, the precise mechanisms are not defined yet [51]. In fact, the liver represents an immunoregulatory hub between the pathogens delivered in the blood and the immune system. The hepatic urea cycle is the main metabolic pathway involved in the detoxification processes, metabolizing ammonia to urea, with a fumarate shunt that connects the urea cycle and the TCA cycle [52]. Consequently, fumarate can be converted to aspartic acid and α-ketoglutaric acid. Also, our hypothesis relies on the fact that the urea cycle is strictly connected with pathways related to amino acids and polyamines metabolism, representing the central core of global nitrogen metabolism. Nitrogen metabolism was found from our data being present between the top-enriched pathways in all mild, moderate, and severe conditions. In addition, we found in moderate and severe COVID-19 patients increased levels of ornithine as the main metabolite of the urea cycle, and also increased levels of aspartate and glutamate, which are linked to the cycle. What is more, ornithine and glutamate showed positive correlation with lactic acid, suggesting that these disturbances may be strictly interconnected with the overall damage induced by SARS-CoV-2 infection, in part including oxygen imbalance and tissue injury. Accordingly, increased levels of glutamic acid were found to positively correlate with anion gap values in severe COVID-19 [42]. Beside its role in the neurotransmission, an important immunomodulator function has been addressed to the glutamate, finding several glutamate receptors on the surface of *T*-cells and glutamate transporters in antigen presenting cells such as dendritic cells and macrophages [42,53].

Remarkably, it has been recently proven that virus infection is able to disturb the hepatic urea cycle and alter the systemic metabolism through the activation of the interferon type I signaling to suppress virus-specific CD8^+^ *T*-cell responses. This led to down-regulation of the enzymes of urea cycle, including ornithine transcarbamylase that converts ornithine to citrulline, suggesting that the hepatocytes are reprogrammed during the infection, showing increased degradation of arginine and accumulation of ornithine, finally limiting aspartate consumption by the urea cycle [54]. In the context of coronaviruses infection, the modulation of the liver metabolism and the urea cycle may act as endogenous immunoregulative mechanisms during SARS-CoV-2 infection and pathology.

Finally, in addition to these regulatory mechanisms, the dysregulation of ornithine metabolism may increase the synthesis of polyamines. In particular, the enzyme ornithine decarboxylase converts ornithine into putrescine, which is in turn converted into spermidine, and then into spermine by the enzymes spermidine synthase and spermine synthase, respectively. Our metabolomics analysis identified spermidine and spermine as slightly increased in the serum of COVID-19 patients. Since the key role of polyamines has been established for several viral processes, such as infection, structural assembling, and genome replication, targeted approaches to block polyamine synthesis have been investigated as potential broad-spectrum antiviral strategies [55]. Such approaches may be taken into account also in the view of combatting SARS-CoV-2 infections.

Globally, our correlation analyses revealed that the highest number of associations with the levels of proinflammatory cytokines mostly involved amino acids and amino acid-related metabolites, but also bile acids, carboxylic acids, and acylcarnitines. In particular, we found a positive correlation between asparagine, isoleucine, leucine, and valine with TNF-α, proline with IL-17 A and IL-17 RA, threonine with IL-26, and a negative correlation of tryptophan with IL-26. Recently, it was found that circulating proinflammatory cytokines levels strongly correlated with amino acids involved in arginine metabolism, tryptophan metabolism, as well as nucleic acid metabolism [18]. Intriguingly, we found a correlation of IL-17 A with xanthine, a purine base that was up-regulated in moderate and severe patients. Xanthine derivative compounds such as caffeine, theophylline, and theobromine have been employed in the treatment of respiratory diseases, cardiovascular diseases, and cancer [56,57,58]. Particularly, caffeine is known to improve the pulmonary functions used as treatment of the apnea of prematurity in preterm infants [59]. The xanthine-derived drug pentoxifylline has shown to have an immunomodulatory role by inducing the downregulation of TNF-α and other inflammatory cytokines in pulmonary diseases and chronic heart failure. Moreover, the effect of pentoxifylline has been demonstrated to treat fibrotic lesions by immunomodulation and reduction of inflammation processes [60]. Since upregulated xanthine (and eventually xanthine-derived compounds) is strongly associated with increased lactic acid levels correlating with the hypoxic state induced by SARS-CoV-2 infection, pentoxifylline and xanthine-derived metabolites may be employed in view of adjuvant treatments of COVID-19 to handle respiratory symptoms, taking advantage from their immunomodulatory and anti-inflammatory properties [61].

## 4. Materials and Methods

### 4.1. Patients

A cohort of 52 COVID-19 patients confirmed by a positive RT-PCR test for SARS-CoV-2 on a nasopharyngeal swab, and 9 control donors with a negative RT-PCR test for SARS-CoV-2 were recruited at one of the following hospitals of Campania region (Italy): Department of Clinical Medicine and Surgery–Section of Infectious Diseases, University Hospital Federico II, Naples; Department of Infectious Disease and Infectious Urgencies–Division of Respiratory Infectious Disease, Cotugno Hospital, AORN dei Colli, Naples, Pathophysiology and respiratory rehabilitation-1 utsir COVID. The 52 COVID-19 patients had a median age of 58 years and were distributed as 36/52 males (70%) and 16/52 females (30%). The distribution of the control group was 40% males and 60% females, with a median age of 46 years. The classification of the COVID-19 cohort was performed on the basis of a seven-point ordinal scale, as explained elsewhere [30]. Then, such patients were assembled in three major groups according to their clinical features, and categorized as mild (*n* = 20), moderate (*n* = 16), and severe (*n* = 16). Serum samples were gathered at hospital admission and stored at −80 °C until metabolomics analysis was performed. All the methods and the experimental procedures were accomplished according to the relevant guidelines and regulations included in the n°191/20 protocol approved by the Ethics Committee at the University of Naples Federico II. The current study was carried out in accordance with the Declaration of Helsinki, and did not include human subjects under the age of 18 years. Each patient (and/or legal guardian) gave fully informed consent for the participation to the research and the use of their biological samples for research purposes.

### 4.2. Sample Preparation and Metabolomics Analysis

The analysis of the serum of COVID-19 patients was performed by targeted metabolomics using tandem mass spectrometry (MS/MS) [27,29,62]. Globally, the analysis performed allowed the quantification of 630 metabolites. Of these, 483 lipids have been analyzed independently and the results obtained on the serum lipidome of COVID-19 patients published previously [30]. In the current work, we have analyzed 146 metabolites (Supplementary Table S1), partitioned as the following: 106 small molecules, including amino acids (AA) (20 molecules), AA related (30 molecules), bile acids (14 molecules), fatty acids (12 molecules), biogenic amines (9 molecules), carboxylic acids (7 molecules), hormones (4 molecules), indoles derivatives (4 molecules), and alkaloids, amine oxides, cresols, vitamins and cofactors (6 molecules), and 40 lipids, including acylcarnitines (40 molecules). The remaining class of carbohydrates that includes the sum of hexoses (1 molecule) was discarded from both the analyses. Metabolome analysis was carried out, following the protocols of MxP^®^ Quant 500 kit (Biocrates Life Sciences AG, Innsbruck, Austria), on a Triple Quad™ 5500 + System–QTRAP^®^ Ready (AB Sciex, Framingham, MA, USA) coupled to a 1260 Infinity II HPLC (Agilent, Santa Clara, CA, USA) for the liquid chromatography (LC). In detail, 10 µL of patient serum were pipetted onto a 96-well extraction plate containing the positions for blanks, PBS, calibrants, and quality controls (QC), and dried under nitrogen stream. Then, each sample was incubated for 1 h with 50 µL of 5% phenyl isothiocyanate (PITC) solution to derivatize amino acids and biogenic amines and dried again. Metabolites were extracted with 300 μL of 5 mM ammonium acetate in methanol in the shaker (30 min, 450 rpm) and eluted in a new 96-well plate by centrifugation. For LC analysis, 150 µL of each extract were diluted with an equal volume of HPLC-grade water, while 10 µL of each extract were diluted for flow injection analysis (FIA) with 490 µL of FIA solvent (provided by Biocrates). After dilution, LC-MS/MS and FIA-MS/MS measurements were carried out to target and quantify by multiple reaction monitoring (MRM) small molecules and lipids, respectively. Data were generated using the Analyst software v.1.7.1 (AB Sciex, Framingham, MA, USA) and further processed for calculating metabolites concentrations using the MetIDQ™ Oxygen software (Biocrates Life Sciences AG, Innsbruck, Austria).

### 4.3. Metabolomics Features Selection and Statistical Analyses

The concentrations of 146 serum metabolites were calculated, and three of them were excluded for carrying invalid values, for a total of 143 quantified metabolites. The metabolomic dataset was analyzed by multivariate statistical analysis using MetaboAnalyst 5.0 software [63,64,65]. The concentration values of the molecules identified in the metabolome of COVID-19 patients and controls sera were imputed to remove missing values, log2-transformed and auto scaled. The normalized dataset was used to perform PLS-DA (Partial Least Squares-Discriminant Analysis), VIP (Variable Importance in Projection), and pattern correlation analyses. The heatmaps were produced using non-normalized individual and average concentrations values in all the groups of patients. In addition, univariate statistical analysis was performed using GraphPad Prism 9.0 (San Diego, CA, USA). In particular, volcano plot analysis was employed to compare each COVID-19 metabolomic dataset at different degree of severity with the control (CTRL) group. After log2 transformation of metabolomic concentrations, unpaired *t*-test with Welch correction was applied. The False Discovery Rate (FDR) approach was selected for the statistical significance of the difference (FDR = 1%), applying the two-stage step-up method of Benjamini, Krieger, and Yekutieli. For each comparison, the relative abundance was calculated as the difference of the log2-transformed means of COVID-19 metabolites and CTRL ones. Differentially abundant metabolites were selected using the following criteria: (i) difference values larger than ± 0.5 and (ii) FDR < 0.01, reported as −log10(*q*-value) > 2.

To measure the strength of association between the 143 quantified metabolites and the levels of the cytokines TNF-α, IL-6, IL-10, IL-17 A, IL-17 RA, and IL-26 in COVID-19 patients, the Spearman correlation was performed computing the *r* value for the metabolites against each cytokine dataset. A confidence interval at 95% was chosen for the statistical significance. The metabolites passing the D’Agostino & Pearson normality and/or lognormality tests were correlated with lactic acid levels applying the Pearson correlation. To detect significant associations within each disease group independently, a confidence interval at 95% was chosen for the statistical significance. Then, the correlating metabolites found significant in all the three disease groups, but which did not show significant correlation also in the control group, were selected and graphed. Correlation analyses were performed using GraphPad Prism 9.0 software.

### 4.4. Metabolic Pathway Analysis

Metabolite set enrichment analysis (MSEA) and pathway analysis were combined to detected significant disturbed metabolic pathways in mild, moderate, and severe patients compared to the control group using MetaboAnalyst 5.0 software. MSEA was performed using the quantitative enrichment analysis (QEA) tool that used the normalized quantitative metabolomics dataset to generate a list of biologically meaningful terms. Then, MSEA analysis was integrated with pathway analysis, which combined the *p*-values obtained from the QEA and the pathway impact values from the pathway topology analysis, selecting the KEGG as pathway library. The software and the KEGG database were not able to match the corresponding names for many acylcarnitines, which were automatically excluded from the analysis. Only significant enriched pathways with FDR < 0.01 were presented. Finally, Euler–Venn analysis was performed using InteractiVenn software to show the relationship between all the significant pathways identified in mild, moderate, and severe COVID-19 patients [66,67].

## 5. Conclusions

Mass spectrometry-based targeted metabolomics analysis performed on the serum of COVID-19 patients allowed us to define a clear signature of the effects of SARS-CoV-2 infection that includes augmented levels of lactic acid in all the disease groups. In particular, moderate and severe patients showed the major dysregulations in metabolite levels, with only little differences between these two groups. Energy production and amino acid metabolism pathways resulted largely dysregulated, suggesting that COVID-19 has a strong impact on the metabolism. Our metabolomics data may provide reasonable indication of liver metabolism injury, suggesting a plausible alteration of carbon and nitrogen metabolism in affected patients.

## Figures and Tables

**Table 1:** List of the differentially abundant metabolites and the respective log2 difference values in the MILD vs. CTRL, MODERATE vs. CTRL, and SEVERE vs. CTRL comparisons.

Metabolite	Difference		
	MILD	MODERATE	SEVERE
β-Alanine	-	0.9	0.9
Arachidonic acid	-	1.5	1.0
Aspartate	-	2.1	1.6
C18:1	-	0.7	-
C5:1	-	−2.5	−1.6
Choline	-	1.6	1.4
Deoxycholic acid	-	−2.9	-
DHEAS	-	-	−2.5
Glutamate	-	3.3	2.9
Hippuric acid	-	−2.5	-
Lactic acid	1.5	2.0	2.2
Ornithine	-	1.0	1.0
Phenylalanine	-	1.4	1.1
Serine	-	0.7	-
Serotonin	-	-	−0.6
Succinic acid	-	0.5	-
Trigonelline	-	−1.8	-
Xanthine	-	2.1	1.4

Metabolites were ordered alphabetically.

**Table 2:** MSEA and pathway analysis in COVID-19 patients. Results of the significant (FDR < 0.01) enriched pathways according to KEGG database.

Enriched Metabolic Pathway	FDR (<0.01); Impact Score		
	MILD	MODERATE	SEVERE
Glycolysis/Gluconeogenesis	1.7 × 10^−5^; 0.0	5.9 × 10^−8^; 0.0	1.5 × 10^−7^; 0.0
Pyruvate metabolism	1.7 × 10^−5^; 0.0	5.9 × 10^−8^; 0.0	1.5 × 10^−7^; 0.0
d-Glutamine and d-Glutamate metabolism	6.0 × 10^−4^; 0.5	2.0 × 10^−5^; 0.5	9.3 × 10^−6^; 0.5
Nitrogen metabolism	6.0 × 10^−4^; 0.0	2.0 × 10^−5^; 0.0	9.3 × 10^−6^; 0.0
Pyrimidine metabolism	6.8 × 10^−4^; 0.0	5.9 × 10^−6^; 0.0	3.5 × 10^−5^; 0.0
Purine metabolism	0.00102; 0.03	3.4 × 10^−4^; 0.03	9.8 × 10^−5^; 0.03
Phenylalanine, tyrosine and tryptophan biosynthesis	-	1.5 × 10^−7^; 1.0	0.00135; 1.0
Phenylalanine metabolism	-	2.2 × 10^−6^; 0.4	0.00528; 0.4
Arginine biosynthesis	-	2.2 × 10^−6^; 0.5	1.8 × 10^−5^; 0.5
Aminoacyl-tRNA biosynthesis	-	5.9 × 10^−6^; 0.2	1.1 × 10^−5^; 0.2
Alanine, aspartate and glutamate metabolism	-	5.9 × 10^−6^; 0.6	5.9 × 10^−6^; 0.6
Glyoxylate and dicarboxylate metabolism	-	1.9 × 10^−5^; 0.2	5.9 × 10^−6^; 0.2
beta-Alanine metabolism	-	2.2 × 10^−5^; 0.45	0.00135; 0.45
Glutathione metabolism	-	6.4 × 10^−5^; 0.1	0.00329; 0.1
Pantothenate and CoA biosynthesis	-	2.7 × 10^−4^; 0.02	0.00528; 0.02
Nicotinate and nicotinamide metabolism	-	0.00498; 0.0	0.00504; 0.0
Porphyrin and chlorophyll metabolism	-	0.00570; 0.0	0.00474; 0.0
Arachidonic acid metabolism	-	0.00191; 0.3	-
Arginine and proline metabolism	-	0.00221; 0.5	-
Propanoate metabolism	-	0.00529; 0.0	-
Selenocompound metabolism	-	0.00753; 0.0	-

Enriched metabolic pathways were ordered according to decrescent FDR in MILD patients. The pathway impact score was shown for each enriched metabolic pathway.

## Supplementary Materials

The following are available online at https://www.mdpi.com/article/10.3390/ijms22179548/s1.
